# Dynamic Mitochondrial Proteome Under Polyamines Treatment in Cardiac Aging

**DOI:** 10.3389/fcell.2022.840389

**Published:** 2022-03-15

**Authors:** Hao Zhang, Meng Yan, Ting Liu, Peiling Wei, Nannan Chai, Lingxu Li, Junying Wang, Xue Yu, Yan Lin, Bintao Qiu, Yajun Zhao

**Affiliations:** ^1^ Department of Pathophysiology, Key Laboratory of Cardiovascular Medicine Research, Harbin Medical University, Harbin, China; ^2^ Department of Pathology, The First Affiliated Hospital of Soochow University, Soochow University, Suzhou, China; ^3^ College of Nursing, Medical School of Chifeng University, Chifeng, China; ^4^ Department of Nephrology, The Second Affiliated Hospital of Hainan Medical University, Haikou, China; ^5^ Department of Medical Technology, Beijing Health Vocational College, Beijing, China; ^6^ Department of Pathophysiology, Qiqihar Medical University, Qiqihar, China; ^7^ Medical Research Center, Peking Union Medical College Hospital, Chinese Academy of Medical Sciences, Beijing, China

**Keywords:** aging, heart, polyamines, mitochondria, proteome, rat

## Abstract

Age-related alteration of mitochondria causes impaired cardiac function, along with cellular and molecular changes. Polyamines can extend the life span in mice. However, whether polyamines can affect the dynamic mitochondrial proteome, thereby preventing age-related changes in cardiac function and cardiac aging, remains unclear. In this study, we found that spermine (Spm) and spermidine (Spd) injection for 6 weeks could prevent 24-month-old rats heart dysfunction, improve mitochondrial function, and downregulate apoptosis. Using iTRAQ tools, we identify 75 mitochondrial proteins of statistically significant alteration in aging hearts, which mainly participate in important mitochondrial physiological activity, such as metabolism, translation, transport, apoptosis, and oxidative phosphorylation. Moreover, four proteins of differential expression, pyruvate dehydrogenase kinase (PDK4), trifunctional enzyme subunit alpha (HADHA), nicotinamide nucleotide transhydrogenase (NNT), and Annexin6, which were significantly associated with heart aging, were validated by Western blotting. *In vitro*, we further demonstrated polyamines could retard cardiomyocytes aging through downregulating the expression of PDK4 and thereby inhibiting cell apoptosis. In summary, the distinct mitochondrial proteins identified in this study suggested some candidates involved in the anti-aging of the heart after polyamines treatment, and PDK4 may provide molecular clues for polyamines to inhibit apoptosis and thus retard aging-induced cardiac dysfunction.

## Introduction

Aging is a degenerative process associated with reduction of physiological function and is the most important risk factor for developing cardiovascular disease. During aging, alteration of mitochondria trigger a wide spectrum of physiological variation, including 1) increased disorganization of mitochondrial structure, 2) decline in mitochondrial oxidative phosphorylation (OXPHOS) function, 3) accumulation of mtDNA mutation, 4) increased mitochondrial production of reactive oxygen species (ROS), and 5) increased extent of oxidative damage to DNA, proteins, and lipids (Lee and Wei, 2012; Jang et al., 2018). Cardiac aging is an intrinsic process that results in impaired cardiac function, along with cellular and molecular changes. The mechanism of cardiac aging is sophisticated, including autophagy, ubiquitin-mediated turnover, apoptosis, mitochondrial quality control, and cardiac matrix homeostasis (Quarles et al., 2015). In these processes, mitochondria have a key role because cardiac function is tightly associated with an energetically demanding process. In the last few decades, several investigations have revealed the relevance of mitochondria and oxidative stress both in cardiac aging and in the development of cardiac diseases, such as heart failure, cardiac hypertrophy, and diabetic cardiomyopathy (Martín-Fernández and Gredilla, 2016). ROS is mainly produced by mitochondria *in vivo*, and increases during aging (Bartke, 2008; Quan et al., 2020). The accumulation of ROS leads to mtRNA mutation, which could develop cardiomyopathy to congestive heart failure (Kujoth et al., 2007). Moreover, evidence suggests that the structure of mitochondria in the heart is broken during the aging process (Dai and Rabinovitch, 2009; Liang and Gustafsson, 2020).

Polyamines (PAs) are small aliphatic ploycations, existing in all living organisms, with most common members, spermine (Spm) and spermidine (Spd) (Haddock Russell, 1980; Wallace, 2009). Polyamines play multiple roles in the cellular process, such as stabilizing and remodeling chromosomes, stimulating cell proliferation, and protecting cells under different types of stress (Miller-Fleming et al., 2015). Polyamines act as ROS scavengers under physiological pH (Fujisawa and Kadoma, 2005). By a number of studies, the mechanism of polyamines function in stress tolerance is illustrated by modulating homeostasis of ROS due to their direct, or indirect, roles in regulating antioxidant systems or suppressing ROS production (Liu et al., 2015). The opening of the mitochondrial permeability transition pore (mPTP) in isolated rat heart and liver mitochondria were reported to be inhibited by polyamines (Lapidus and Sokolove, 1994; Zhao et al., 2007). In addition, the transport of mitochondrial calcium and mitochondrial biogenesis were also influenced by polyamines (Elustondo et al., 2015; Wang et al., 2020a). Eisenberg et al. revealed that Spm could enhance longevity by suppression of necrosis and induction of autophagy (Eisenberg et al., 2009). A recent study showed that naturally Spd has beneficial effects on cardiac aging in mice and high-salt-induced congestive heart failure in rats due to an underling mechanism (Eisenberg et al., 2009; Eisenberg et al., 2016; Muñoz-Esparza et al., 2019). Our previous study has shown that exogenous Spm restores the intracellular polyamines pool and confers cardioprotective effect against ischemia and reperfusion (IR) injury through stabilizing the cell membrane, scavenging free radicals, and preventing intracellular free calcium elevation (Zhao et al., 2007).

In a post-genomics age, proteomics provides powerful tools to give a global view of protein profiles in cells or tissues. In research of the aging field, Chakravarti et al. (2008) discovered differential proteomic patterns for aging mouse hearts by 2DE and LC-MS/MS. Several proteomic research also reported the alternation in mitochondria during aging, involved in OXPHOS and mPTP proteins suppression, calcium homeostasis alterations, and metabolites catabolism breakdown (Fu et al., 2009; Stauch et al., 2015; Pfanner et al., 2019). However, there is seldom a systematic report for mitochondrial alternation under effect of polyamines during the aging process. In this study, we worked to reveal the effect of polyamines during the age-associated myocardial pathological changes by proteomic strategy, aiming to find the clue to the mechanism of heart aging.

## Methods

### Animal Care and Polyamines Administration

The male Wistar rats aged 3 months (young, Y) and 24 months (old, O) were purchased from the Animal Center of Harbin Medical University. All procedures conformed to the Guide for Care and Use of Laboratory Animals published by the China National Institutes of Health, and the study protocols involving the use of animals were approved by the Institutional Animal Center of Harbin Medical University. The old rats were randomly divided into old control (O, *n* = 8), spermine (Spm, *n* = 8), and spermidine (Spd, *n* = 8) groups, they were injected intraperitoneally with saline, spermine (2.5 mg/kg), and spermidine (10 mg/kg) for 6 weeks, respectively, young rats injected with saline were assigned to young control (Y, *n* = 8). Spm and Spd were purchased from Sigma-Aldrich (St. Louis, MO, United States), and the optimal concentration used was performed as previously described (Zhang et al., 2017; Chai et al., 2019; Wang et al., 2020a; Wang et al., 2020b). From each experimental group we euthanized six rats to collect hearts. All animals were housed under conditions of constant temperature and humidity, a standard light/dark cycle, and free access to standard rodent chow and water.

### Echocardiography

Transthoracic echocardiography with Doppler ultrasound techniques was used to assess left ventricular systolic (LVIDs) and diastolic (LVIDd) parameters and function. Briefly, rats were anaesthetized by inhalation of 1.5%–2% isoflurane. The measurement was performed with Acuson CV-70 (Siemens) using standard imaging planes by an observer blinded to the treatment. The parameters were obtained in M-mode tracings at the papillary muscle level or pulse doppler evaluation of the bicuspid valve and averaged using at least five continuous cardiac cycles.

### Immunohistochemistry and Morphological Analyses

The IHC, Masson, and TUNEL staining were performed on formalin-fixed and paraffin-embedded cardiac tissue sections. For IHC staining of β-galactosidase, the tissue sections were deparaffinized, rehydrated, and antigen retrieval, and were incubated with anti-β-galactosidase (Santa Cruz, United States) overnight, and then were incubated with biotinylated secondary antibody (Zhongshan, China) followed by avidin-biotin peroxidase complex according to the manufacturer’s instructions. Finally, tissue sections were incubated with Diaminobenzidine (Sigma) until a brown color was developed. For Masson staining, the sections were visualized at 40× magnification, and volume fraction of collagen (VFC) fibril structures were quantified from three sections per heart. Results were averaged from five high power random fields from each section. The apoptotic myocytes were detected by TdT mediated dUTP nick end-labeling (TUNEL) assay using a Cell Death Detection Kit (Roche, Germany) according to the manufacturer’s instructions. Briefly, the cardiac tissue sections were incubated with a TUNEL reaction mixture containing TdT and fluorescein-dUTP after permeability treatment. The TUNEL signal was detected by an anti-fluorescent antibody conjugated with alkaline phosphatase, a reporter enzyme, which catalytically generates a colored product. Three slides from each block were evaluated for percentage of apoptotic cells. Four slide fields were randomly examined with 200× magnification. A total of 100 cells were counted in each field.

### Cardiac Mitochondria Isolation

Rat heart mitochondria were isolated at 4°C by differential centrifugation using a mitochondrial isolation kit (Beyotime Biotechnology, Inc.). Briefly, the rat left ventricular tissue was washed and added 10 times the isolation buffer A. The tissue was finely minced with scissors and then homogenized in the ice-cold buffer, the following processing we did as previously described (Wang et al., 2014). At last, the homogenate was centrifuged at 10,000 g and the pellet was resuspended in ice-cold isolation buffer B prior to the ultrastructural analysis and mitochondrial respiration function assessment.

### Transmission Electron Microscopy

Isolated mitochondria: The cardiac mitochondrial homogenate was centrifuged at 3000 g and the pellet was fixed in 3% glutaraldehyde for 2–4 h at 4°C, then washed, dehydrated, embedded in resin, and sectioned. The sections were stained with uranyl acetate and lead nitrate and then visualized with an electron microscope (S4800 Hitachi, Tokyo, Japan).

### Measurement of Mitochondrial Respiration Function

Mitochondrial oxygen consumption was measured using a Clark-type oxygen electrode (Hansatech Instruments, Norfolk, United Kingdom) in mitochondrial respiration buffer. Pyruvate (5 mM) and malate (5 mM) were used as substrates for complex I-containing mitochondria at a final concentration of 500 µg protein/mL. Mitochondrial State 3 respiration, State 4 respiration, respiratory control ratio (RCR), ADP/O ratio, and proton leakage were measured as previously described (Wang et al., 2014).

### Measurement of Antioxidant Enzyme Activity

Superoxide dismutase (SOD) activity in cardiac tissue was measured using commercial kits (SOD: A001-3-1; Jiancheng Bio. Institute, Nanjing, China) with a spectrophotometer (Perkin- Elmer, Norwalk, CT, United States). Protein concentration was measured using the bicinchoninic acid method (Pierce, Rockford, United States) with bovine serum albumin (BSA) as a standard.

### Measurement of ROS

ROS generation was measured by dihydroethidium (DHE) staining assay (Cat No. S0063, Beyotime, China). Briefly, frozen tissue sections of cardiac tissues were incubated with 5 μmol/L DHE at 37°C for 30 min, then washed with PBS three times and analyzed using a fluorescence microscope. Images were taken with an Olympus FluoView FV1000 fluorescence microscope (Olympus Optical Co., Ltd., Takachiho, Japan) at an excitation wavelength of 535 nm, and the maximum emission wavelength is 610 nm.

### Mitochondria Protein Extraction, Trypsin Digestion, and iTRAQ Labeling

The mitochondria extracted in rat cardiomyocytes were divided into Y, O, Spm, and Spd. Each sample was mixed by multiple parallel samples, and each group of technologies was repeated once. All the reagents and buffers needed for iTRAQ labeling and cleaning were purchased from Applied Biosystems (Foster City, CA, United States). The iTRAQ labeling protocol was referred to the manufacturer’s instructions. In brief, mitochondrial proteins were extracted by 8 M Urea supplemented with 10 mM DTT, pH 8.5 (Amesco St. Louis, MO, United States) and concentration was determined by the Bradford assay. Proteins were dissolved, denatured, alkylated, and digested with trypsin (Promega) at 37°C overnight. To label peptides with iTRAQ reagent, 1 unit of label (defined as the amount of reagent required to label 100 μg protein) was thawed and reconstituted in 150 μl of isopropanol. The digestions from CSM_100_ and SBM were labeled with 114 and 116 iTRAQ reagents, respectively, and this reaction was repeated with 115 and 117 iTRAQ reagents again to guarantee the accuracy of quantitation. Moreover, a strong cation exchange (SCX) column was used for (Applied Biosystems) separating the mixed peptides.

### Analysis by Q-Exactive Mass Spectrometer and Data Processing

The fractionated peptides were analyzed by a Q-Exactive mass spectrometer fitted with a nano-liquid chromatography system. The eluent was introduced directly to a Q-Exactive mass spectrometer via EASY-Spray ion source. As for data processing, the Proteome Discoverer software 1.3 was employed to interpret raw data and protein quantitation. The rat protein database was downloaded from Uniprot database and combined with the reversed sequences and sequences of widely spread contaminants, such as human keratins for false discovery rate (FDR) control. Finally, the FDR was set to 0.01 to get high confidence results.

### Validation of iTRAQ Results by Western Blotting

The mitochondrial protein concentration was measured by BCA Protein Assay Reagent Kit (Beyotime, Nantong, China). Samples containing total mitochondrial protein were separated by 10% (w/v) SDS-PAGE and transferred onto a PVDF membrane (Millipore, Bedford, MA, United States) (details are in Western Blotting Assay section). The following antibodies were used: Antibodies for PDK4 (Cat# 12949-1-AP), HADHA (Cat# 10758-1-AP), Annexin 6 (Cat# 12542-1-AP), NNT (Cat# 13442-2-AP), and COX-IV (Cat# 66110-1-Ig) were obtained from Proteintech (Wuhan, Hubei, China). The secondary antibody (alkaline phosphatase-conjugated anti-rabbit IgG) was from Promega Corporation (Madison, WI, United States). The intensities of the protein bands were quantified using a Bio-Rad ChemiDocTM EQ densitometer and Bio-Rad Quantity One software (Bio-Rad, Hercules, United States).

### Bioinformatics Analysis

Gene Ontology (GO) provides a controlled vocabulary to describe the attributes of genes and gene products in any organism and is applied for cardiovascular research (Lovering et al., 2009). Combining the GO and Uniprot database, the function and localization was annotated for the differentially expressed proteins. The protein-protein interaction networks (PPI) were analyzed in STRING online database v11.5 (http://www.string-db.org/).

### Isolated and Culture of Neonatal Cardiomyocytes

Neonatal rat cardiomyocytes (NRCMs) were isolated and cultured by standard methods as previously described (Wei et al., 2016). Three days after being seeded, these cardiomyocytes were divided randomly into the following. Control group: cells cultured under normal incubation conditions. H_2_O_2_ group (H_2_O_2_-induced cell senescence group): cardiomyocytes were pre-treated with 40 μM H_2_O_2_ for 4 h, then cultured for 24 h the same as the control group. H_2_O_2_-Spm, H_2_O_2_-Spd, and H_2_O_2_-DAC groups: cardiomyocytes were pre-treated with 40 μM H_2_O_2_ for 4 h, then incubated with 20 μM Spm, 10 μM Spd, or 1 mM dichloroacetate (DCA) for 24 h, respectively. H_2_O_2_-Spd-DFMO group: before the cells were treated with 40 μM H_2_O_2_, they were pre-incubated with the 5 mM difluoromethylornithine (DFMO, an inhibitor of ODC) for 1 h. The followed treatment was the same as the H_2_O_2_-Spd group. Both DFMO and DCA were purchased from Sigma-Aldrich (St. Louis, MO, United States), and the optimal concentration applied was performed as previously described (Wang et al., 2020a).

### Senescence-Associated β-Galactosidase Staining

Cellular senescence was determined by SA-β-gal staining. Staining was performed using SA-β-gal staining kit (Beyotime, Haimen, China) according to the manufacturer’s guidelines. Positive staining was evaluated after 12–16 h incubation at 37°C in a CO_2_-free atmosphere. The blue stained cells from 10 different fields were counted with results presented as a percentage of positive cells.

### JC-1 Staining

JC-1 (Beyotime Biotech, C2006), a sensitive fluorescent probe for ΔΨm, was used to measure the ΔΨm of cardiomyocytes. In healthy cells with high mitochondrial ΔΨm, JC-1 forms aggregates with intense red fluorescence; in apoptotic or unhealthy cells with low ΔΨm, JC-1 remains in the monomeric form, which shows green fluorescence. According to the manufacturer’s directions, the cells were incubated with JC-1 staining solution (5 μg/ml) for 20 min at 37°C and then washed twice with Tyrode standard solution. The images were viewed and scanned under laser confocal microscopy (OLYMPUS, FV1000, Japan) at 488 nm excitation and 530 nm emission for green, and at 543 nm excitation and 590 nm emission for red. The ratio of red to green fluorescence was calculated as ΔΨm. Mitochondrial depolarization is indicated by a reduction in the red/green fluorescence intensity ratio. Image processing and analysis were performed with ImageJ software.

### Statistics

All statistical analyses were performed using SPSS 17.0 (SPSS, Chicago, IL, United States). Values are expressed as mean ± SEM. Mann-Whitney U Test was used for comparing between two groups for non-normally distributed data. Comparisons between the mean of three or more groups were done by one-way ANOVA. Correlation analysis was performed by linear regression. Differences were considered significant at *p* < 0.05.

## Result

### Polyamines Protected Cardiac Function in the Aging Heart

**FIGURE 1:**
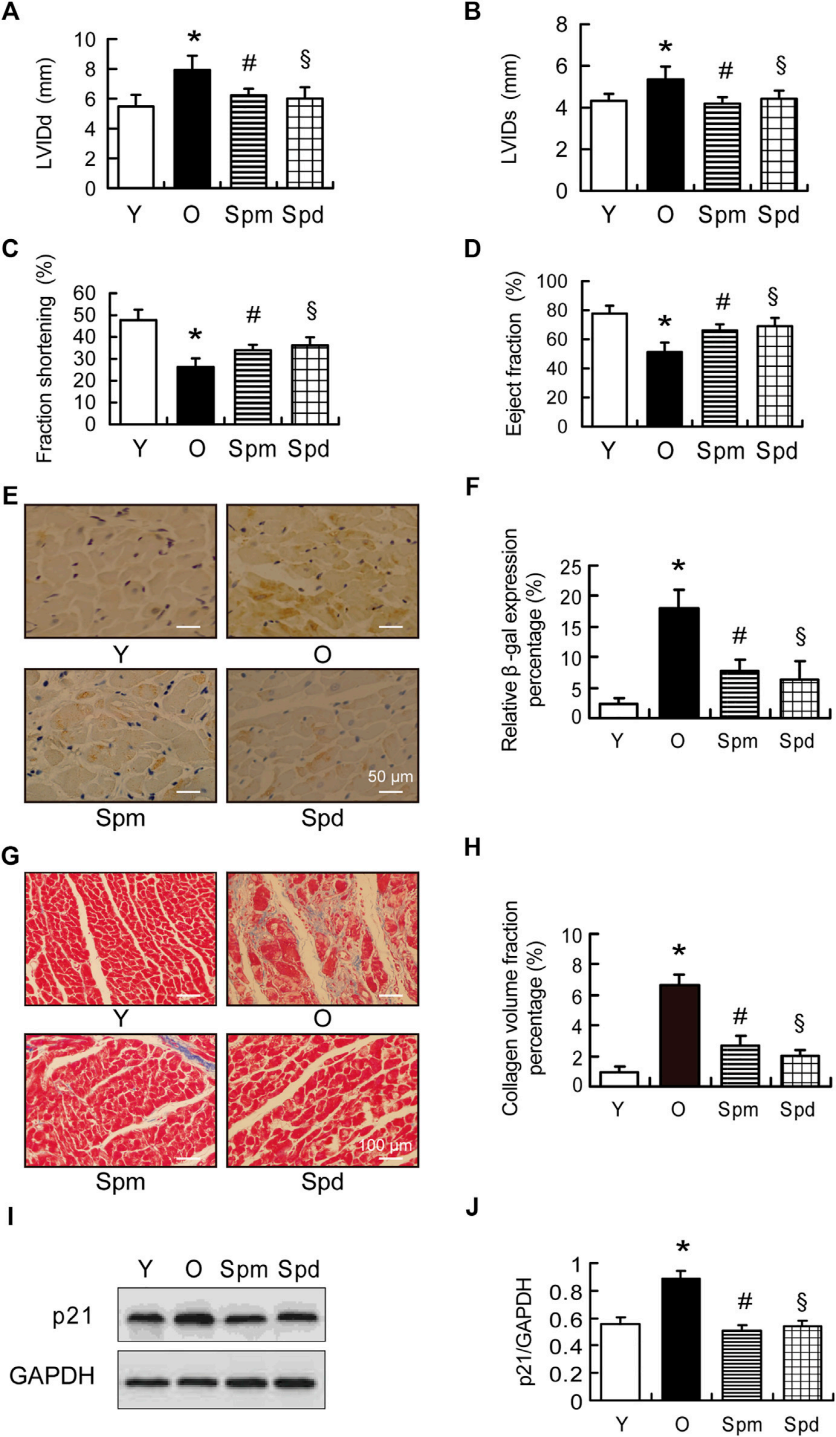
Cardiac function in polyamines-treated old rats. **(A,B)** Left ventricular internal diameter at end-diastole (LVIDd) and left ventricular internal diameter at end-systole (LVIDs) in indicated groups (*n* = 8). **(C)** Fractional shortening (FS) and **(D)** eject fraction (EF) were measured by tissue Doppler imaging of the mitral annulus (*n* = 8). **(E)** The expression of β-gal was assayed by immunohistochemical (IHC) staining, staining is shown, scale bar = 50 μm. **(F)** Quantitative analysis of β-gal positive cells (*n* = 8). **(G,H)** Masson staining and collagen volume fraction represent interstitial fibrotic areas in the left ventricle midwall sections in indicated groups, scale bar = 100 μm (*n* = 8). **(I,J)** The expression of p21 was assayed by Western blotting (WB), GAPDH is used as the internal loading control (*n* = 6–8). Mean ± SEM; *p*-values correspond to one-way ANOVA; *p < 0.05 vs. Y; ^§^ *p* < 0.05, and ^#^ *p* < 0.05 vs. O.

To investigate the effect of polyamines on age-related cardiac changes, we first examine the cardiac function between young (3-months-old) and old (24-month-old) rat hearts with or without Spm and Spd supplementation. Echocardiographic evaluation revealed a significant increase in the left ventricular internal diameter at end-diastole (LVIDd) and end-systole (LVIDs), as well as a decrease in the shortening fraction (FS) and ejection fraction (EF) in old rats compared with young rat hearts. Spm and Spd injection reversed aging-induced unfavorable changes in cardiac function (Figures 1A–D). Furthermore, SA-β-gal was stained in the cytosol of the cardiomyocyte, the aged heart showed significantly higher β-gal staining when compared to the young heart. Spm and Spd supplementation overtly decreased aging-induced β-gal staining elevation in old hearts (Figures 1E,F). Disordered collagen fiber network was also observed in old rat hearts, and collagen volume fraction (CVF) was higher than young rat hearts. After Spm or Spd complement, CVF of old rat hearts was decreased to a greater extent (Figures 1G,H). Similarly, a significant increase of p21 was seen in old hearts compared with the young, and the age-related expression of p21 was significantly attenuated by Spm and Spd treatment in the aged heart (Figures 1I,J). These findings suggested that Spm and Spd treatments can effectively inhibit the decrease of age-related heart function and cardiomyocytes aging.

### Polyamines Improved Mitochondrial Function in the Aging Heart

**FIGURE 2:**
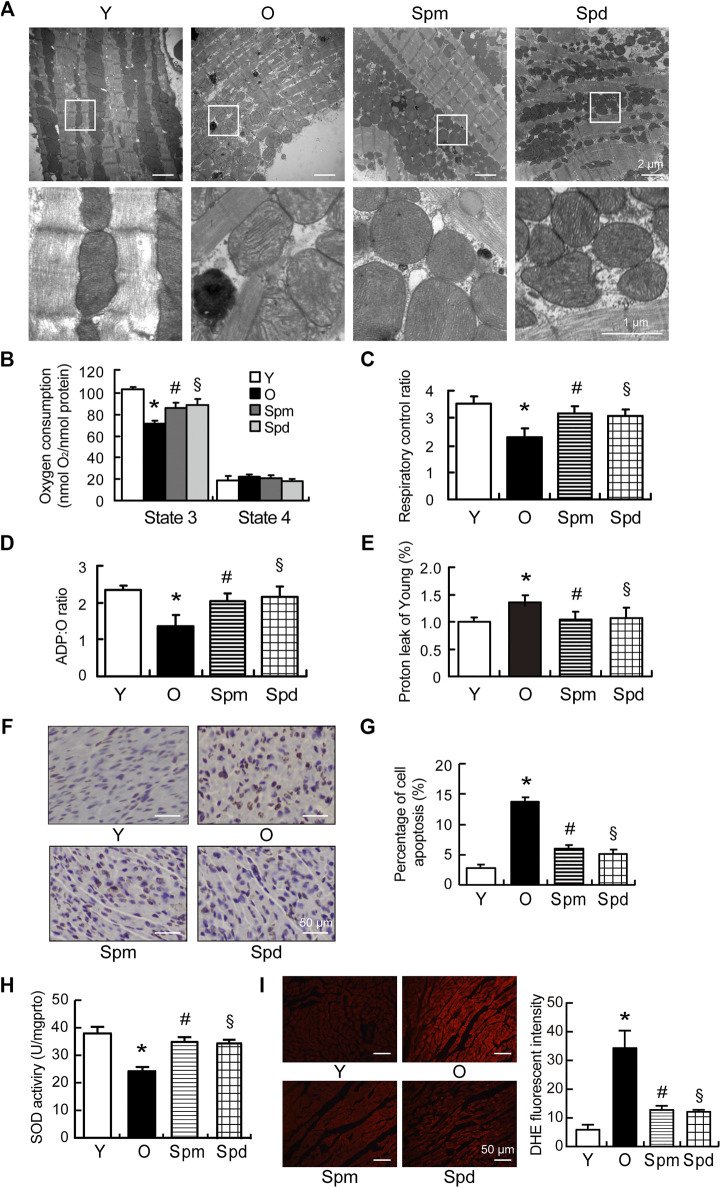
Mitochondrial function in polyamines-treated old rats. **(A)** Ultrastructure changes of cardiomyocytes under transmission electron microscope (TEM), scale car = 2 μm (*n* = 3). **(B–E)** Mitochondrial oxidative phosphorylation (OXPHOS) efficiency was evaluated in the rat myocardium. Measurements included **(B)** mitochondrial oxygen consumption States 3 and State 4; **(C)** respiratory control rate (RCR); **(D)** ADP/O ratio and **(E)** proton leakage. Respiration was induced with pyruvate/malate (5 mM each) as energizing substrates and ADP (200 μM) to initiate State 3 respiration (*n* = 8). **(F)** Nuclei with brown staining indicate TUNEL-positive cells, while the normal nuclei are blue, scale bar = 50 μm. **(G)** Quantitative results of the percentage of TUNEL-positive nuclei to total nuclei in different groups (*n* = 5). **(H)** SOD activities of cardiac tissues were detected by SOD activity assay (*n* = 5). **(I)** Representative images of ROS changes in myocardial tissues detected by DHE staining assay, scale bar = 50 μm. Fluorescence intensity analysis of ROS changes in myocardial tissues (*n* = 5). Mean ± SEM; *p*-values correspond to one-way ANOVA; *p < 0.05 vs. Y; ^§^ *p* < 0.05, and ^#^ *p* < 0.05 vs. O.

To evaluate the mitochondrial function under polyamines treatment, transmission electron microscope (TEM) was applied to observe the changes of mitochondrial morphology in cardiomyocytes. Mitochondria are longitudinally arranged in the spaces between myofibrils, and mitochondrial cristae were clearly observed in young cardiomyocytes. However, the myocardial fiber structure was disordered, mitochondria were swollen, and mitochondrial matrix density was decreased in aged rats. In contrast, the polyamines treatment heart showed clear cardiac sarcomeres and a dense mitochondrial matrix, only a few mitochondria were showed mildly swollen (Figure 2A). Furthermore, we isolated cardiac mitochondria and measured respiratory function in rats, including the respiratory rates of State 3 and State 4, the respiratory control rates (RCR), ADP/O ratio, and proton leakage using pyruvate/malate as substrates. Proton leakage (H^+^) was measured as oligomycin-inhibited State 3 respiration, which was normalized to young hearts. We found that the State 3 respiratory rate, RCR, and ADP/O ratio were significantly decreased, and proton leakage was increased in the old myocardial mitochondria compared with the young group (*p* < 0.05 for all). State 3 respiratory rate, RCR, and ADP/O ratio were higher, and the oxidative phosphorylation independent proton leakage was lower in cardiac mitochondria of polyamines treated old rat hearts than that in normal old hearts. However, there were no significant differences in the mitochondrial State 4 respiratory rate among any of the experimental groups (Figures 2B–E). As we know, mitochondria play a significant role in energy metabolism, redox balance, and apoptosis regulation (Jang et al., 2018). Thus, we evaluated the effect of polyamines on apoptosis in aging hearts by TUNEL assay. The results showed a significant increase in the number of TUNEL-positive cardiomyocytes in cardiac tissue from old hearts compared with young hearts, and polyamines supplementation inhibited such an increase (Figures 2F,G). Furthermore, Spm and Spd treatment significantly attenuated oxidative stress in aging myocardium, evidenced by decrease in SOD activity (Figure 2H) and reduction in the fluorescent intensity of DHE (Figure 2I). These data suggest that polyamines improve mitochondrial respiration function through inhibiting oxidative stress and preventing cell apoptosis in myocardium of the aged rat.

### Mitochondrial Proteomic Alteration After Polyamines Treatment in the Aging Heart

**FIGURE 3:**
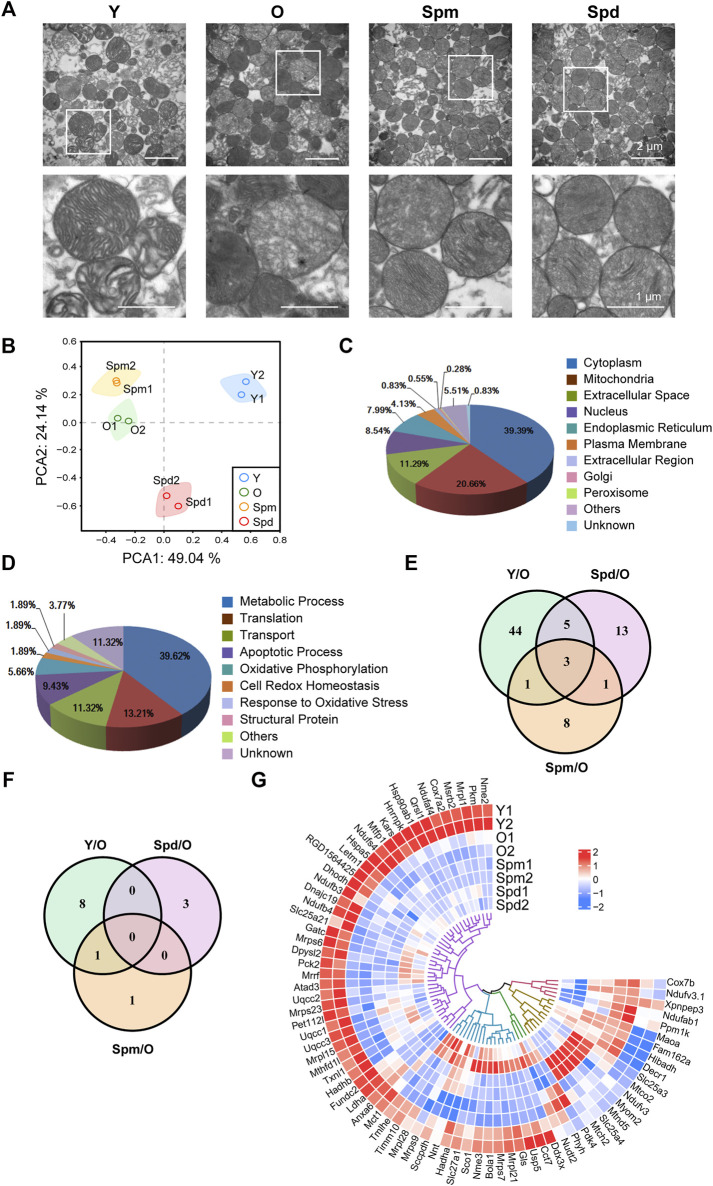
Bioinformatics analysis of cardiac mitochondrial proteome. **(A)** Ultrastructure changes of isolated cardiac mitochondria under TEM. A sizable proportion of extracts were mitochondria with intact membrane and cristae, scale bar = 2 μm (*n* = 3). **(B)** Principal component analysis (PCA) was built based on 364 protein measurements. Axes show clear differentiation among samples, a different colored circle represents each sample group (*n* = 3). **(C)** The distribution of identified proteins localization by GO. **(D)** The distribution of function of identified proteins by GO. **(E)** Venn diagram showing the overlap between Y vs. O, Spm vs. O, and Spd vs. O in 75 mitochondrial proteins. **(F)** Venn diagram showing 13 proteins which expressed more than 1.5-fold. **(G)** Heatmap of protein levels of identified proteins (*n* = 3).

To evaluate potential correlations between polyamines and mitochondrial, we isolated mitochondria from four different groups, including Y, O, Spm, and Spd. We first observed the ultra-structure changes of isolated mitochondria with TEM, and found the mitochondria isolated from young hearts showed intact mitochondrial inner and outer membranes, cristae arranged neatly and matrix dense, while the mitochondria from old hearts showed matrix loose, ruptured inner and outer membrane, cristae decreasing or disappearance, and some of mitochondria changed to vacuole. In contrast, Spm and Spd complement significantly retarded the ultra-structure changes of the aged heart mitochondria. Intact mitochondrial membranes, dense matrix, and more clear mitochondrial structure were also observed after polyamines treatment (Figure 3A). Then, the isolated mitochondria were labeled with iTRAQ regent and analyzed by Q-Elite. Principal component analysis (PCA) of the processed datasets was performed to visualize the clustering trends among the samples selected. In the score plot of PCA, the different groups Y, O, Spm, and Spd were well differentiated (Figure 3B). In total, 36,251 tandem mass spectra were captured and searched against the Uniprot rat protein database using stringent matching criteria, resulting in the confident identification of 1355 gene products. According to the report ions, 364 differentially expressed proteins were identified, of which 95 proteins with more than 1.5 folds were selected (Supplementary Table S1). These proteins represented the difference among Y vs. O, Spm vs. O, and Spd vs. O. There were 53 upregulated (ratio < 1.50) proteins and 19 downregulated (ratio < 0.67) proteins between young and old rats. As for Spm injection group, there were 6 upregulated proteins and 18 downregulated proteins. Whereas 9 upregulated proteins and 13 downregulated proteins were identified in the Spd injection group. Additionally, some differentially expressed proteins were identified in both Spm or Spd groups, such as pyruvate dehydrogenase kinase (PDK4), protein Hbb-b1, and protein RT1-A1. Owing to contamination during mitochondria isolation, the localization information of all the differentially expressed proteins should be annotated. To do so, GO, a well-established knowledge database, was employed to give systematic annotation for these proteins. As a result, there are 75 proteins localized in mitochondria, which is about 20% in all 364 differentially expressed proteins (Figure 3C; Supplementary Table S2). While proteins localized in cytoplasm and extracellular space may be contaminants in mitochondria isolation. The function of identified proteins was also annotated by GO, which includes metabolism, translation, transport, apoptosis, and oxidative phosphorylation, and most of these items are tightly related with mitochondrial physiological function (Figure 3D). Among all these 75 mitochondrial proteins, 13 proteins were differentially expressed more than 1.5-fold, Venn diagrams represent the distribution of protein identifications for each group compared to the old (Figures 3E,F). Furthermore, heatmap analysis of the 75 mitochondrial proteins revealed notable differences between Y, O, Spm, and Spd, and some proteins expressed in the polyamines treatment group were remarkably close to the young group (Figure 3G). In summary, our results suggested that polyamines treatment might have an effect on alleviating cardiac aging.

### Effects of Polyamines on Mitochondrial Biological Processes in the Aging Heart

**FIGURE 4:**
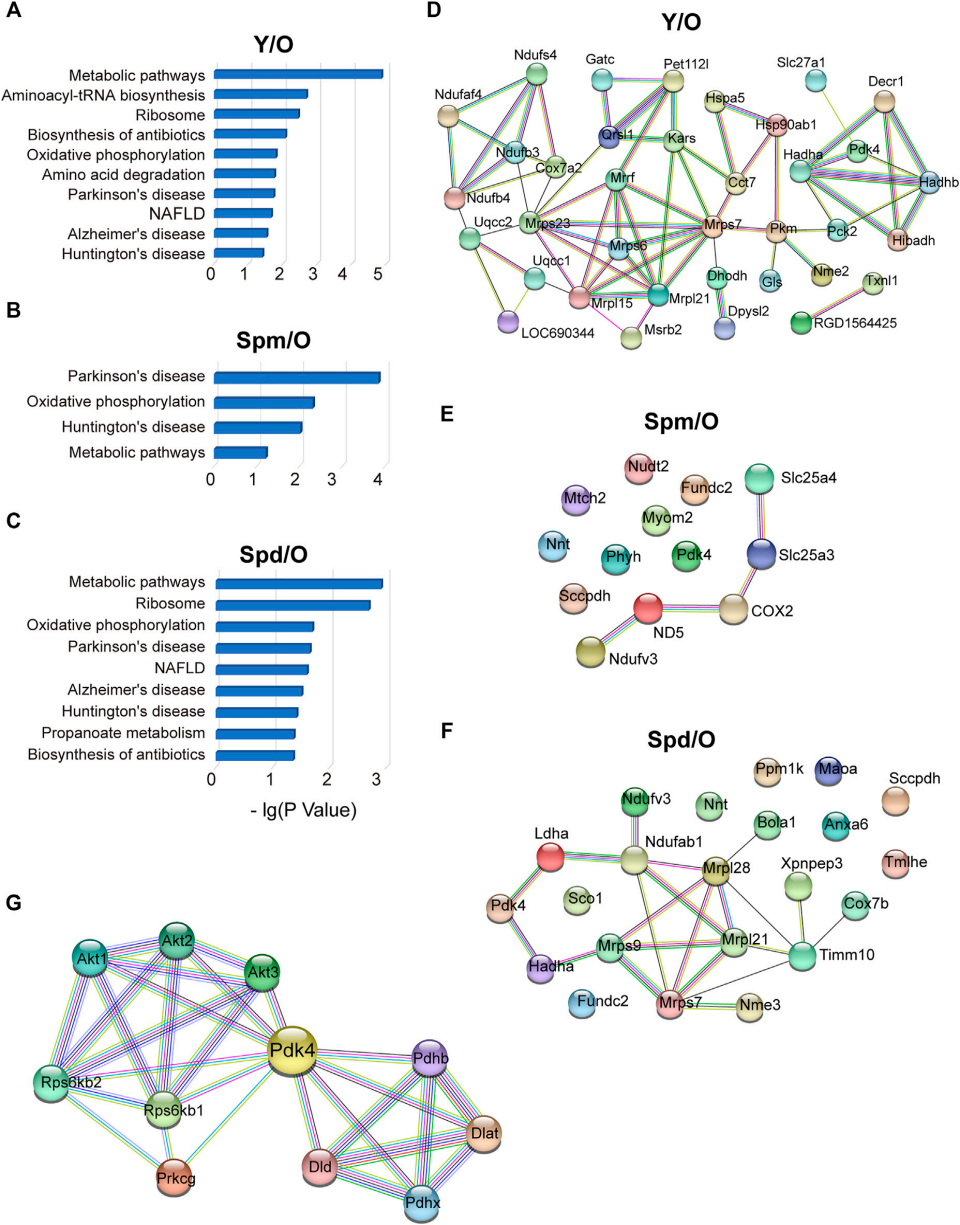
Protein–protein interactions of cardiac mitochondrial proteome. **(A–C)** Canonical pathways regulated in Y vs. O, Spm vs. O, and Spd vs. O groups. **(D–F)** STRING PPI analysis of differentially expressed proteins in Y vs. O, Spm vs. O, and Spd vs. O. **(G)** Construction of interaction network of PDK4 by the String database.

Next, the MetaCore tool was used to study canonical pathways differentially regulated by Spm and Spd treatment in aged rat mitochondria, respectively. Aging gave the most alteration on the metabolic pathway, while the biosynthesis process and oxidative phosphorylation were also altered when comparing young and old rat mitochondria (Figure 4A). Crucial polyamines-altered biological processes include metabolic pathway, oxidative phosphorylation, and aging-related diseases (Figures 4B,C), which means polyamines treatment has an effect on aging-related metabolic pathways and diseases. To gain more insight into the biological functions and molecular characteristics of the differentially expressed proteins, protein–protein interactions (PPI) networks were constructed by the STRING database, which has a collection of known and predicted PPI, including direct (physical) and indirect (functional) associations. We examined 53 mitochondrial proteins differentially expressed in young compared to old, 22 proteins followed the Spd treatment and 13 proteins followed the Spm treatment vs. old (Figures 4D–F). More interestingly, there were conspicuous PPI among polyamines and aging. Thus, we speculate that polyamines treatment may play a significant role in regulating the mitochondrial-related metabolic mechanism and aging progress. Specially, PDK4 was obviously downregulated both in Y vs. O (Y:O = 0.50104) and Spm vs. O (Spm:O = 0.490375). PDK4 also has a decreasing trend after Spd treatment (Spd:O = 0.736708). Of note, there are certain differences in the canonical pathway and PPI networks of mitochondria proteome after Spm and Spd treatment. Spd treatment detected more differentially expressed mitochondrial proteins, indicating that Spd has a stronger regulatory effect on mitochondria in aging rats. Furthermore, we analyzed the interaction network of PDK4 from the String database, the members of which include RPS6KB1, RPS6KB2, DLD, DLAT, PDHX, PDHB, PRKCG, AKT1, AKT2, and AKT3 (Figure 4G). Some of them have already been reported to regulate the aging process, but mitochondrial PDK4 regulation by polyamines stimulation in aging is still not clear. Thus, we paid our attention to these mitochondrial proteins for further biological verification.

### Validation Analysis of Significant Changes in Protein Abundance Induced by Polyamines in the Aging Heart

**FIGURE 5:**
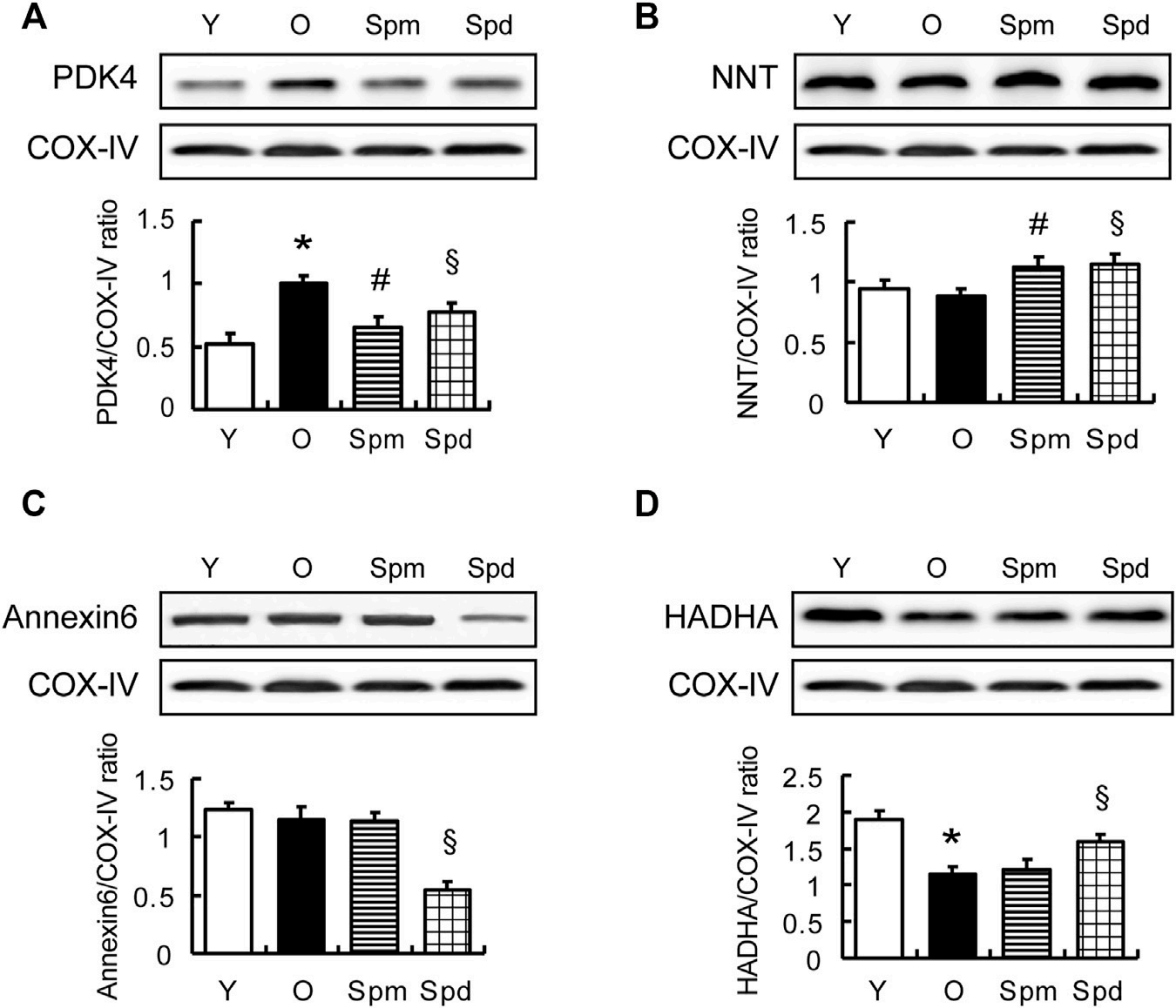
Validation of differentially expressed proteins. **(A–D)** WB analysis of PDK4, NNT, Annexin 6, and HADHA protein level in myocardial tissue of Y, O, Spm, or Spd-treated rats (top); histogram shows the expression levels of the four proteins as determined by densitometric analysis (bottom), mitochondrial markers protein (COX IV) is used as the internal loading control (*n* = 6). Mean ± SEM; *p*-values correspond to one-way ANOVA; *p < 0.05 vs. Y; ^§^ *p* < 0.05, and ^#^ *p* < 0.05 vs. O.

To confirm the result of the iTRAQ experiment, 4 differentially expressed proteins, PDK4, mitochondrial trifunctional enzyme subunit α (HADHA), Annexin6, and nicotinamide nucleotide transhydrogenase (NNT) were chosen for validation. In addition to PDK4 and NNT, Annexin6 is closely associated with the cristae in the inner membrane of mitochondria (Rainteau et al., 1995) and HADHA catalyzes the last three steps of mitochondrial beta-oxidation of long-chain fatty acids (Liang et al., 2018; Adeva-Andany et al., 2019). Results showed the relative protein level of PDK4, NNT, Annexin6, and HADHA, as normalized to COX-IV. As expected, the results of Western blotting were in line with our bioinformatic analysis of mitochondrial proteome. Compared with the old hearts, PDK4 was downregulated and NNT was upregulated in both Spm and Spd treated groups, and PDK4 differed significantly (Figures 5A,B). However, the HADHA level was upregulated and Annexin 6 was downregulated in only the Spd treated group, but no response to Spm supplementation (Figures 5C,D). Hence, our results suggested that the protected effects of polyamines in aging cell mitochondria were associated with mitochondrial PDK4 protein.

### Polyamines Retard Cell Aging Through Downregulating PDK4 in NRCMs

**FIGURE 6:**
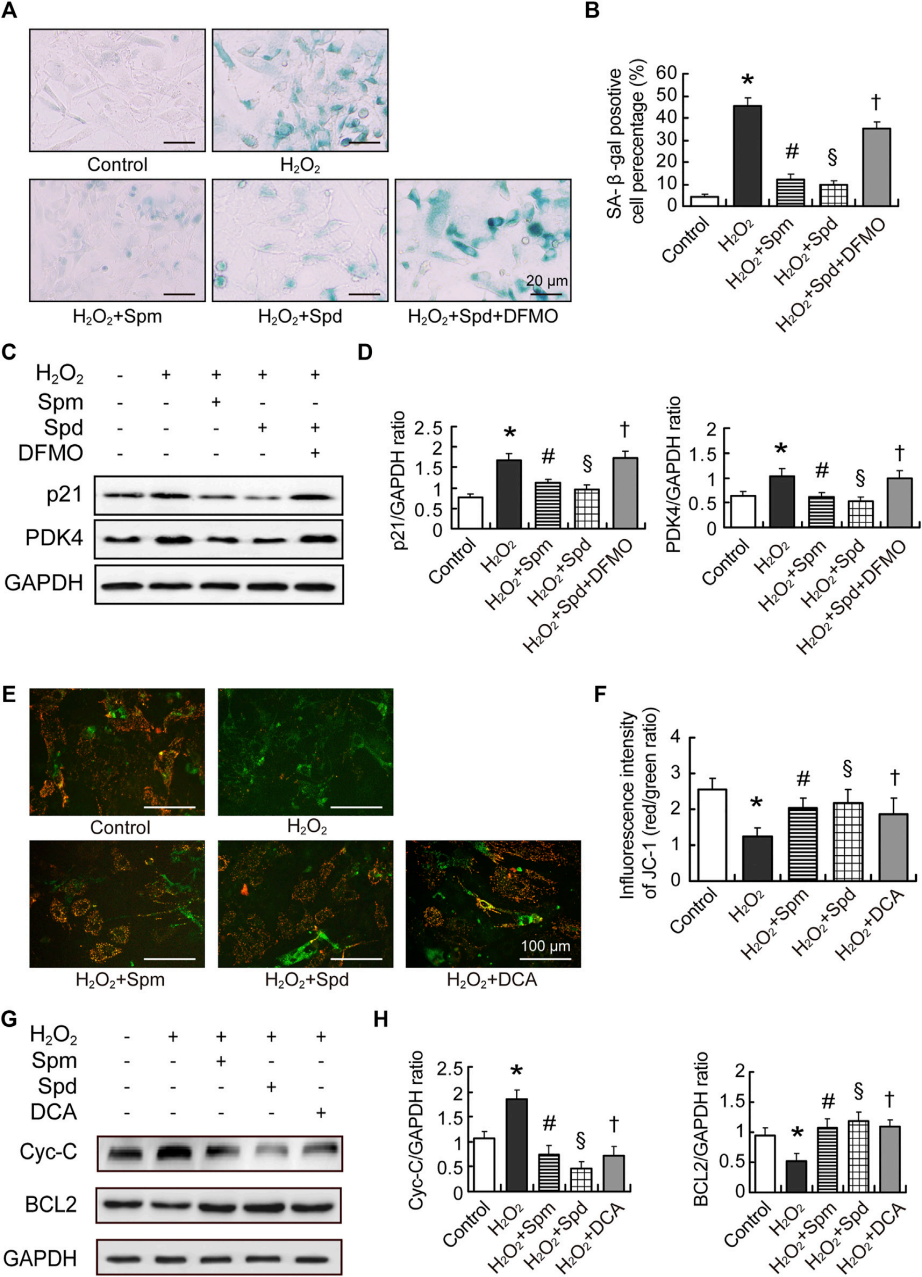
Polyamines inhibited cardiac aging through downregulating PDK4. **(A)** Representative images showing the morphological changes and senescence-associated- β-galactosidase (SA-β-gal) activity in cardiomyocytes treated with H_2_O_2_, H_2_O_2_-Spm, H_2_O_2_-Spd, and H_2_O_2_-Spd-DFMO, scale bar = 20 μm. **(B)** Quantitative analysis of SA-β-gal-positive cells percentage (*n* = 4). **(C)** The expression of p21 and PDK4 protein evaluated by WB analysis. **(D)** Quantitative analysis of p21 and PDK4 level normalized to GAPDH (*n* = 4–6). **(E)** Representative images of JC-1staining in cardiomyocytes treated with H_2_O_2_, H_2_O_2_-Spm, H_2_O_2_-Spd, and H_2_O_2_-DAC (an inhibitor of PDK4), respectively. Red fluorescence represents JC-1 aggregates formed in normal cells with high Δψm, whereas green fluorescence represents JC-1 monomers in cells with low Δψm, scale bar = 100 μm. **(F)** Quantitative analysis of the shift of mitochondrial red fluorescence to green fluorescence among groups. The ratio of red to green fluorescence was calculated as ΔΨm (*n* = 4). **(G)** Cytochrome-C (Cyt-C) and BCL2 protein levels in cardiomyocytes. **(H)** Quantitative analysis of Cyt-C and BCL2 level normalized to GAPDH (*n* = 4–6). Mean ± SEM; *p*-values correspond to one-way ANOVA; *p < 0.05 vs. control; ^§^ *p* < 0.05, and ^#^ *p* < 0.05 vs. H_2_O_2_; ^†^ *p* < 0.05 vs. H_2_O_2_-Spd.

Following our observation that PDK4 level reduced after polyamines supplementation, we further investigated the correlation between PDK4 and polyamines in aging cardiomyocytes. Primary culture neonatal rat cardiomyocytes (NRCMs) were induced aging by H_2_O_2_. Cell aging was identified by SA-β-gal staining and p21 expression level. We found that aging cardiomyocytes showed enlarged volume, flattened morphology, and more SA-β-gal positive cells. The expression of p21 was increased obviously more than that in the control group, while Spm and Spd treated groups showed a decrease in SA-β-gal positive cells and p21 expression. Difluoromethylornithine (DFMO, an inhibitor of ODC) treatment abolished the Spd-mediated decrease (Figures 6A–D). We further observed a significant increase in the expression of PDK4 after H_2_O_2_ induction, and polyamines treatment lowered the expression. In addition, DFMO abolished the Spd-induced decrease of PDK4 expression (Figures 6C,D). These data suggested that polyamines retard cell aging partly through inhibiting PDK4 in NRCMs. As we know, a hallmark of cardiac aging is total number of cardiomyocytes decreased by apoptosis (Broughton et al., 2018). To elucidate the role of Spm and Spd in H_2_O_2_-induced cell apoptosis, we evaluated the loss of Δψm by JC-1 staining (Figures 6E,F) and assessed Cytochrome-C (Cyt-C) and BCL2 expression by Western blotting (Figures 6G,H). JC-1 assay showed that compared to the control group, Δψm in the H_2_O_2_ group was notably compromised, presenting as a large area of green fluorescence. Regarding protein expression, the expression level of Cyt-C was elevated accompanying declining levels of BCL2 expression after H_2_O_2_ pre-treatment. As expounded in Figure 6, pre-treating with 10 μM Spm or 20 μM Spd before the cell incubation with H_2_O_2_ protected the cells against apoptosis, showing Δψm and BCL2 level elevation and Cyt-C expression reduction. DCA, an inhibitor of PDK4, was utilized to assess the influence of PDK4 on H_2_O_2_-induced apoptosis. Accordingly, DCA treatment got the same trend with Spm and Spd treated groups (Figures 6E–H). Hence, the effect of polyamines acted as a PDK4 inhibitor to markedly prevent cardiomyocytes apoptosis after H_2_O_2_-induction.

## Discussion

The aim of the current study was to identify alterations in mitochondrial proteome under polyamines (Spm or Spd) stimulation with a high-throughput quantitative iTRAQ approach during the age-associated myocardial pathological changes, which may contribute to resisting cardiac aging and delaying senility. Here, we established the map of differentially expressed mitochondrial proteome of the aging heart under polyamines stimulation, in which potential candidates were discovered and given further analysis.

In this study, we first analyzed differentially expressed mitochondrial proteins in young and old rat hearts. We found the difference between old and young mitochondria is more than the other two groups, including 3 downregulated (MAOA, PDK4, FAM162A) and 6 upregulated (NDUFB4, PET112-like, UQCC2, UQCC3, HSP90, FUNDC2) mitochondrial proteins, which indicates mitochondrial function is complicated and regulated by multiple pathways in the mechanism of aging. These differentially expressed proteins participate in many biological processes, such as metabolic process, translation, transport, apoptotic process, and oxidative phosphorylation. It is well established that metabolic dysfunction including fatty acid (FA) metabolism and oxidative phosphorylation reduction is associated with aging and age-related diseases (Stanley et al., 2005; Hu and Liu, 2014). In addition, the biological functions of many other proteins have been suggested in mitochondrial proteomics. Ubiquinol-Cytochrome-C reductase complex assembly factor 2 (UQCC2, Y:O = 1.540) is much higher in young rats than in old ones, which is demonstrated to modulate mitochondrial oxygen consumption, and regulate the respiratory chain activity and ATP production (Cambier et al., 2012). Accordingly, we proposed that aging causes remodeling of the cardiac mitochondrial proteomics which could suppress mitochondrial metabolism and function, which leads to enhanced oxidant production, oxidative injury and the activation of oxidant signaling for cell death, and finally increase the sensitivity of the aging heart to stress. While the maintenance of mitochondrial proteomic homeostasis could be beneficial to preserve heart function in aged hearts.

Next, we observed the effect of Spm and Spd on mitochondrial proteome and related these to changes in mitochondrial function in aged hearts. We found that, after 6 weeks treatment with Spm launched at old age rat, 6 proteins were upregulated and 18 proteins were downregulated, while 9 proteins were upregulated and 13 proteins were downregulated in the Spd injection group. NADH-ubiquinone oxidoreductase chain (MTND5, Spm:O = 1.749) was elevated significantly, and MTND5 is the core subunit of the mitochondrial membrane respiratory chain NADH dehydrogenase (Complex I). It was reported that the mutation of MTND5 is associated with cardiomyopathy, which exhibits impairment of mitochondrial complex I assemble, decrease of coupling respiration and adenosine triphosphate (ATP) generation (Marin-Garcia et al., 2000; Ayalon et al., 2013). Therefore, the high level of MTND5 expression in old heart with Spm-treatment suggested Spm may contribute to keep the heart healthy against aging. In contrast to Spm injection, more proteins are differentially expressed in Spd stimulation. Nicotinamide nucleotide transhydrogenase (NNT, Spd:O = 1.571) is a nuclear gene encoded protein located in the mitochondrial inner membrane, which catalyzes mitochondrial transmembrane hydride transfer between NAD(H) and NAD(P)+ to generate NAD(P)H. It has been demonstrated that the NAD+/NADH ratios affect the activity of the sirtuins family, which are potential mediators of the beneficial effects of calorie restriction and mediating lifespan, and an age-dependent loss of gene expression of NNT which linked to mitochondrial dysfunction (Olgun, 2009). Moreover, NNT increases NADPH level, which is important for antioxidant systems, biosynthesis, aging, and apoptosis (Zhang et al., 2021). NNT overexpression can be used as management strategies to decrease the ratio of NADH/NAD+ and aging. Our research showed Spd increased NNT level in old hearts, which might be one of the molecular mechanisms of Spd delaying age-related mitochondrial damage. X-Prolyl Aminopeptidase 3 (XPNPEP3) is mitochondrial protein encoded by nuclear genes, which is conserved through evolution. Research revealed that loss of its ortholog gene Icp55p function increased the chronological lifespan and oxidative stress resistance while decreasing respiratory metabolism in *S. cerevisiae* (Stames and O'Toole, 2013). Thus, the low expression of XPNPEP3 (Spd:O = 0.601) after Spd treatment would lead to apparent benefits such as improved mitochondrial function and increased stress resistance in the aging heart.

Except for proteins that have changed more than 1.5-fold, some proteins also changed moderately after Spm or Spd treatment in the aging heart, such as LDHA (Spd:O = 0.755), SCO1 (Spd:O = 1.33068), SLC25A3 (Spm:O = 1.311), and SLC25A4 (Spm:O = 1.430). LDHA is involved in the sub-pathway that synthesizes (S)-lactate from pyruvate and is a key mediator of anaerobic glycolysis. Small molecule inhibition of LDHA resulted in a switch from aerobic glycolysis to oxidative phosphorylation and this metabolic shift was accompanied by an increase in mitochondrial content and cellular ATP levels in human pluripotent stem cell-derived cardiomyocytes (Hu et al., 2018). In our research, Spd inhibited aging-induced LDHA elevation in rats (Spd:O = 0.755), which indicated Spd might have a potential in promoting cardiomyocytes mitochondrial function. SCO1 is a mitochondrial copper-binding protein adding copper ions to the catalytic core of cytochrome-c oxidase (COX) (Stiburek et al., 2009). However, in Sco1 mutation patients, it exhibits a combined deficiency in COX activity and total copper content (Leary et al., 2013). SCO1 increasing in Spd-treated aging rats (Spd:O = 1.33068) indicates the protected function of Spd in COX activity and cellular copper homeostasis. Adenine nucleotide translocases (ANTs) are protein families, 2 paralogs SLC25A3 (Spm:O = 1.311) and SLC25A4 (Spm:O = 1.430) were both upregulated in our result. ANTs could catalyze the exchange of cytoplasmic ADP with mitochondrial ATP across the mitochondrial inner membrane, playing an essential role for energy metabolism of eukaryotic cells (Cho et al., 2015). SLC25A3 transports inorganic phosphate into the mitochondrial matrix, playing an essential role in the aerobic synthesis ATP (Mayr et al., 2007; Bhoj et al., 2015). As for SCL25A4, it is reported that mutation of SCL25A4 is associated with autosomal recessive myopathy and cardiomyopathy, including cardiac hypertrophy, cardiomyocyte degeneration, and extensive sub-endocardial interstitial fibrosis (Strauss et al., 2013). These results indicated that remodeling of the mitochondrial proteome in the aged rat heart can be partly reversed by exogenous polyamines supplement. Previously, we have demonstrated both Spd and Spm can inhibit the decrease of age-related mitochondrial State 3 respiratory, the increase of ROS production and cell apoptosis, and improved heart function. Therefore, we inferred polyamines prevented the changes of mitochondrial/cardiac function that are associated with aging through maintaining the homeostasis of the mitochondrial proteome. These results were also supported by other’s findings, they demonstrated that Spd and Spm played anti-aging effects through inhibiting oxidative stress and improving mitochondrial function (Fischer et al., 2020; Xu et al., 2020).

Polycationic polyamines Spd and Spm are essential for mammalian cell growth and survival. Dysfunction of them has been implicated in human health and diseases, such as cancer, inflammation, stroke, renal failure, diabetes, cardiovascular disease, cognitive impairment, and other ailments (Amin et al., 2021). Mostly, Spd shares similar biological activities to Spm, but sometimes they showed a diverse mechanism in various pathological and physiological conditions or in the different cells and tissues. For instance, the effect of cardiovascular-protection and anti-aging of both Spm and Spd were performed through inducing autophagy, reducing age-associated oxidative stress, and inhibiting inflammation. The most frequently reported is that the beneficial effects of Spd are causally connected to the capacity of Spd to induce cytoprotective autophagy/mitophagy by modulating the expressions of *Atg* genes, promoting the acetylation of *Atg* genes by inhibiting EP300, and suppressing histone acetylation (Madeo et al., 2018; Ghosh et al., 2020). Spd inhibited aging-associated pathologies, and pro-inflammatory status is closely associated with autophagy inducing, which would lead to improvement of mitochondrial metabolism and respiration as well as proteostasis (Singh et al., 2017). In contrast, Spm supplement prevents aging-associated pathologies, including pro-inflammatory status by inhibiting aging-associated aberrant DNA methylation (Soda, 2020). Spm extends the lifespan more and tends to act as free radical scavengers (Mayr et al., 2007), to quench singlet molecular oxygen, and shield DNA from oxidative damage (Watkins et al., 2011; Dong et al., 2013; Da Pozzo et al., 2017; Smirnova et al., 2018). It is noteworthy that recent research revealed that Spd has a unique role as the precursor of hypusine, a post-translational modification of the eIF5A, which is necessary for this protein to function in protein synthesis. However, no unique role for Spm has been identified unequivocally (Pegg, 2014). In our study, we noticed that Spd affects a wider array of mitochondrial proteins than Spm does in aged hearts. According to the biological functions and molecular characteristics, it seems that Spd treatment influenced mitochondrial protein synthesis and oxidoreductase activity, Spm has an essential role in mitochondrial electron transport and oxidative phosphorylation. The natural Spm and Spd carry nitrogen moieties, which are positively charged under physiological conditions. This feature provides them with an ability to interact with negatively charged nucleic acids and various proteins and influence critical cell functions. The number of positively charged amino groups linked with each PA are the key factors behind the PAs activity. Here, unequal effect between Spd- and Spm-treated on proteomic in the aging heart causes us to speculate that the anti-cardiac aging protection of Spd could be achieved by different mitochondrial mechanisms than Spm. In brief, Spd and Spm play a positive role on anti-cardiac aging by controlling mitochondrial proteomics homeostasis. Further investigation is needed to verify the different mechanisms of Spm and Spd action.

PDK4 is a negative regulator of the glycolytic enzyme pyruvate dehydrogenase (PDH) and cardiac-specific PDK4 overexpressed mice could perturb metabolism and exacerbate calcineurin-induced cardiomyopathy (Zhao et al., 2008; Sin et al., 2015), and the deficiency of PDK4 could attenuate fat accumulation in the livers of mice fed a high-fat diet (Hwang et al., 2009; Tao et al., 2013). In the present study, we demonstrated the significant role of PDK4 in mediating cardiac aging. Our proteomics results showed that PDK4 was downregulated both in young hearts (Y:O = 0.501) and Spm-treated hearts (Spm:O = 0.490). The protein verification experiment also demonstrated a similar trend of PDK4 expression to proteomics results, which means Spm could regulate the PDK4 expression directly and influence the metabolic pathway in old rat hearts. By construction of a PDK4 interaction network, we found PDK4 had a tight relationship with cardiomyocytic aging. According to the interaction network of PDK4 from the String database, DLD is involved in caloric restriction (CR)-preventable mitochondrial ROS in yeast aging process (Tahara et al., 2007), and PDHX reduction is related to aging diseases, such as Alzheimer’s disease, glucose intolerance, and cancer (Moreau et al., 2004; Stacpoole, 2012). There were many reports that indicated that the AKT family had effect on life span and regulated the aging process through eNOS and PI3K pathway (Smith and Hagen, 2003; Eto et al., 2008; Nojima et al., 2013). These results suggested that polyamines retarded cardiomyocytes aging might partly depend on downregulating the expression of PDK4. In a separate cell experiment, we identified Spm and Spd could inhibit the increase of PDK4 expression and cell aging induced by H_2_O_2_ in NRMCs, and the effect was abolished by the polyamines synthesis inhibitor, DFMO. Moreover, we revealed that PDK4 inhibitor DCA played an anti-apoptosis function, which is in line with Spm and Spd action in increasing mitochondrial membrane potential, upregulating Cytochrome-C expression, and downregulating BCL2 expression. All the results suggested that polyamines could promote cell survival against the cardiac aging by improving mitochondrial metabolism and function both from *in vivo* and *in vitro*. PDK4 blocking by Spm and Spd may become a new therapeutic strategy against heart aging. Recent studies supported our conclusion. They reported that PDK4 is almost invariably upregulated in mitochondrial dysfunction-related metabolic diseases. PDK4 deficiency and DCA treatment exert a therapeutic effect on these diseases (Jeon et al., 2021). In addition, knockout of PDK4 and DAC enhanced survival and improves lifespan, the salutary effects dependent on increased metabolic shifting from aerobic glycolysis to oxidative phosphorylation and reduced ROS production in *C. elegans* (Moreau et al., 2004; Mouchiroud et al., 2011; Schaffer et al., 2011). However, Hyyti et al. observed that a marked decrease in PDK4 expression in aged mice, the downregulation of PDK4 may be accounted for by the suppression of the expression of cardiac PPARα(Hyyti et al., 2010). We speculated weakly that the paradoxical results might be associated with the different experiment model used, they performed the experiment in an isolated working mouse heart, and our research was performed in isolated cardiac mitochondrial and isolated cardiomyocytes from rats. Additional studies are needed to assure the changes of PDK4 during the progression of cardiac aging, and to elucidate the exact role of PDK4 in mitochondrial metabolic in aged hearts.

In conclusion, some key proteins were discovered in our findings, which provided valuable insight in possible experimental targets for understanding the effect of polyamines on aging resistance. There are some limitations to this study, that we did not perform experiments with PDK4 siRNA-mediated silencing in aging cardiomyocytes in the current study, we only used DCA to inhibit PDK4. We will further investigate the potential mechanism of anti-aging by polyamine through loss-of-function and gain-of-function mutations in PDK4.

## Data Availability

The mass spectrometry proteomics data have been deposited to the ProteomeXchange Consortium via the PRIDE[31] partner repository with the dataset identifier PXD031270.
