# Supplementary material for: Dynamic Mitochondrial Proteome Under Polyamines Treatment in Cardiac Aging
Source: Front Cell Dev Biol. 2022 Mar 15;10:840389. doi: 10.3389/fcell.2022.840389 (PMC8965055; doi:10.3389/fcell.2022.840389)
Supplement: Supplementary file 1 [file DataSheet1.DOCX]

Supplementary Material

# Supplementary Tables

| Protein Name | Ratio（Y/O） | Ratio（Spm/O） | Ratio（Spd/O） |
| --- | --- | --- | --- |
| Serum albumin | 1.44618 | - | 0.35148 |
| Hemoglobin subunit alpha-1/2 | 0.526798 | 0.392353 | 0.451605 |
| Keratin, type I cytoskeletal 14 | 1.96184 | - | 0.526041 |
| Hemoglobin subunit beta-2 | 0.651335 | 0.441802 | 0.559483 |
| Protein Hbb-b1 | 0.578525 | 0.493922 | 0.582907 |
| Ig gamma-2B chain C region | 0.482179 | 0.733353 | 0.593589 |
| Probable Xaa-Pro aminopeptidase 3 | - | - | 0.601183 |
| Myosin-3 | - | 2.2473 | 0.606572 |
| Cytochrome c oxidase subunit 7B | - | - | 0.617145 |
| Keratin, type II cytoskeletal 5 | 1.71988 | 0.610541 | 0.619069 |
| Apolipoprotein B editing complex 2 | - | - | 0.643375 |
| Protein RT1-A1 | 0.517788 | 0.626441 | 0.644053 |
| Ig gamma-2A chain C region | 0.486935 | 0.642021 | 0.644502 |
| Keratin, type II cytoskeletal 1 | 1.53112 | 0.56511 | 0.686236 |
| Hemoglobin subunit beta-1 | 0.690412 | 0.568699 | 0.709316 |
| Protein Fundc2 | 1.65005 | 0.75845 | 0.730198 |
| Ig lambda-2 chain C region | 0.643812 | 0.7031 | 0.733116 |
| Pyruvate dehydrogenase kinase | 0.50104 | 0.490375 | 0.736708 |
| Keratin, type I cytoskeletal 15 | - | 0.626441 | 0.742485 |
| Uncharacterized protein | 0.587806 | 0.686895 | 0.74402 |
| Protein Adipoq | - | 0.61362 | 0.750096 |
| Amine oxidase A | 0.425642 | - | 0.750781 |
| Uncharacterized protein | 1.50613 | - | 1.32928 |
| Protein RGD1560831 | 1.65251 | - | 1.32945 |
| Protein RGD1309188 | 1.51226 | - | 1.33054 |
| Adenylosuccinate synthetase isozyme 1 | 1.7829 | - | 1.33412 |
| Cytoskeleton-associated protein 4 | 1.70562 | - | 1.3409 |
| Myosin light chain kinase 3 | 1.63607 | - | 1.35328 |
| Protein Ptprm | 1.64785 | - | 1.41598 |
| Prostamide/prostaglandin F synthase | 1.30384 | - | 1.5217 |
| Protein ERGIC-53 | 1.55749 | - | 1.55743 |
| Isochorismatase domain-containing protein 1 | - | - | 1.57052 |
| Nicotinamide nucleotide transhydrogenase | - | 1.37562 | 1.57085 |
| Peptidyl-glycine alpha-amidating monooxygenase | 1.36784 | - | 1.63126 |
| Myosin light chain 4 | 1.71981 | - | 1.65904 |
| Uncharacterized protein | 1.98094 | - | 1.8136 |
| 40S ribosomal protein S26 | 1.40125 | - | 1.81987 |
| Natriuretic peptides A | 1.79671 | - | 3.15637 |
| Protein Kng2 | 0.504246 | 0.500993 | - |
| T-kininogen 1 | 0.563157 | 0.580528 | - |
| Septin-9 | 1.35712 | 0.594675 | - |
| Uncharacterized protein | 0.700223 | 0.648196 | - |
| Alpha-1-acid glycoprotein | 0.713608 | 0.652556 | - |
| NSFL1 cofactor p47 | - | 0.657854 | - |
| Protein Spta1 | - | 0.661221 | - |
| EH domain-containing protein 1 | 0.669969 | 0.763909 | - |
| Apolipoprotein A-II | 1.69373 | 1.3014 | - |
| Elongation factor 1-alpha 2 | 1.94851 | 1.31116 | - |
| Calcium-transporting ATPase | 1.57519 | 1.32432 | - |
| Myosin regulatory light chain 2 | - | 1.56 | - |
| Histone H4 | - | 1.694 | - |
| NADH-ubiquinone oxidoreductase chain 5 | - | 1.74921 | - |
| Myosin-7 | 0.669165 | 1.9191 | - |
| Myosin-6 | 1.52641 | 2.00062 | - |
| Protein NEWGENE_621351 | 0.536054 | - | - |
| Osteoglycin | 0.592721 | - | - |
| Protein FAM162A | 0.60752 | - | - |
| Lumican | 0.63601 | - | - |
| Maleylacetoacetate isomerase | 0.640741 | - | - |
| Dermatopontin (Predicted), isoform CRA_a | 0.6422 | - | - |
| Protein Igkc | 0.661736 | - | - |
| 40S ribosomal protein SA | 1.50016 | - | - |
| Protein Txndc5 | 1.5057 | - | - |
| 60S acidic ribosomal protein P0 | 1.50606 | - | - |
| Protein Myh14 | 1.53246 | - | - |
| Protein LOC688963 | 1.5326 | - | - |
| PET112-like isoform CRA_b | 1.53764 | - | - |
| Ubiquinol-cytochrome-c reductase complex assembly factor 2 | 1.53967 | - | - |
| 40S ribosomal protein S12 | 1.54226 | - | - |
| Ubiquinol-cytochrome-c reductase complex assembly factor 3 | 1.54233 | - | - |
| Protein LOC102551071 | 1.54238 | - | - |
| Eukaryotic translation elongation factor 1 beta 2 | 1.5476 | - | - |
| SPARC | 1.54898 | - | - |
| Sodium/potassium-transporting ATPase subunit beta-1 | 1.54907 | - | - |
| Actin-related protein 2 | 1.54982 | - | - |
| Heterogeneous nuclear ribonucleoprotein M | 1.55674 | - | - |
| 26S proteasome non-ATPase regulatory subunit 2 | 1.55704 | - | - |
| Guanine nucleotide-binding protein subunit beta-2-like 1 | 1.56386 | - | - |
| Oxysterol-binding protein | 1.5639 | - | - |
| Heterogeneous nuclear ribonucleoprotein R | 1.59499 | - | - |
| Keratin, type II cytoskeletal 2 epidermal | 1.5973 | - | - |
| Endoplasmic reticulum aminopeptidase 1 | 1.60087 | - | - |
| Prolyl 4-hydroxylase subunit alpha-1 | 1.60779 | - | - |
| Heat shock protein HSP 90-beta | 1.61332 | - | - |
| Protein Kb15 | 1.63661 | - | - |
| cAMP-dependent protein kinase type I-alpha regulatory subunit | 1.65351 | - | - |
| Sodium/calcium exchanger 1 | 1.66541 | - | - |
| Fusion, derived from t(1216) malignant liposarcoma | 1.68672 | - | - |
| Transthyretin | 1.75443 | - | - |
| Centrosomal protein of 162 kDa | 1.77939 | - | - |
| Peptidyl-prolyl cis-trans isomerase B | 1.79022 | - | - |
| GTP-binding protein SAR1b | 1.84361 | - | - |
| UV excision repair protein RAD23 homolog B | 1.91809 | - | - |
| Four and a half LIM domains protein 2 | 1.96119 | - | - |
| Serine / Cysteine proteinase inhibitor | 2.05707 | - | - |

**Supplementary Table 1. 95** **proteins with more than 1.5-fold changes identified by mitochondrial proteome.**

| Protein Name | Ratio（Y/O） | Ratio（Spm/O） | Ratio（Spd/O） |
| --- | --- | --- | --- |
| Amine oxidase [flavin-containing] A | 0.425642 | - | 0.750781 |
| Pyruvate dehydrogenase kinase | 0.50104 | 0.490375 | 0.736708 |
| Protein | 0.60752 | - | - |
| Phosphate carrier protein | 0.71469 | 1.31118 | - |
| 3-hydroxyisobutyrate dehydrogenase | 0.760878 | - | - |
| 2,4-dienoyl-CoA reductase | 0.765968 | - | - |
| Protein LOC100359687 | 1.30031 | - | - |
| Dihydropyrimidinase-related protein 2 | 1.30161 | - | - |
| Heterogeneous nuclear ribonucleoprotein K | 1.303 | - | - |
| Protein Dnajc19 | 1.30405 | - | - |
| Saccharopine dehydrogenase-like oxidoreductase | 1.30737 | 1.32987 | 1.31468 |
| Glutamyl-tRNA(Gln) amidotransferase subunit A | 1.31131 | - | - |
| Protein Ddx3x | 1.31237 | - | - |
| Long-chain fatty acid transport protein 1 | 1.31255 | - | - |
| Lysine--tRNA ligase | 1.31697 | - | - |
| Methionine-R-sulfoxide reductase B2 | 1.32079 | - | - |
| Protein Cct7 | 1.32546 | - | - |
| Protein LOC100361144 | 1.3311 | - | - |
| Protein RGD1564425 | 1.33786 | - | - |
| Trimethyllysine dioxygenase | 1.33857 | - | 1.42463 |
| Protein Uqcc1 | 1.33925 | - | - |
| 28S ribosomal protein S7 | 1.33968 | - | 1.37108 |
| Mitochondrial ribosomal protein L15 | 1.34271 | - | - |
| Dihydroorotate dehydrogenase (quinone) | 1.34552 | - | - |
| NADH dehydrogenase [ubiquinone] 1 alpha subcomplex assembly factor 4 | 1.34678 | - | - |
| NADH dehydrogenase [ubiquinone] iron-sulfur protein 4 | 1.35228 | - | - |
| Pyruvate kinase PKM | 1.35895 | - | - |
| Monocarboxylate transporter 1 | 1.36053 | - | - |
| LETM1 and EF-hand domain-containing protein 1 | 1.36243 | - | - |
| Mitochondrial protein 18 kDa | 1.36283 | - | - |
| Protein Pck2 | 1.37667 | - | - |
| Cytochrome c oxidase subunit 7A2 | 1.38215 | - | - |
| Mthfd1l protein | 1.39556 | - | - |
| Glutaminase kidney isoform | 1.3997 | - | - |
| RCG58764 | 1.4004 | - | - |
| 78 kDa glucose-regulated protein | 1.40458 | - | - |
| Trifunctional enzyme subunit beta | 1.41038 | - | - |
| Mitochondrial ribosomal protein L21 | 1.42449 | - | 1.34349 |
| Ubiquitin carboxyl-terminal hydrolase | 1.42602 | - | - |
| Nucleoside diphosphate kinase B | 1.42859 | - | - |
| ATPase family AAA domain-containing protein 3 | 1.42935 | - | - |
| Trifunctional enzyme subunit alpha | 1.43501 | - | 1.32086 |
| Mitochondrial ribosomal protein S23 | 1.44452 | - | - |
| Glutamyl-tRNA(Gln) amidotransferase subunit C | 1.4461 | - | - |
| Ribosome-recycling factor, mitochondrial | 1.45927 | - | - |
| Mitochondrial 2-oxodicarboxylate carrier | 1.46094 | - | - |
| Thioredoxin-like protein 1 | 1.46848 | - | - |
| Protein LOC688963 | 1.5326 | - | - |
| PET112-like | 1.53764 | - | - |
| Ubiquinol-cytochrome-c reductase complex assembly factor 2 | 1.53967 | - | - |
| Ubiquinol-cytochrome-c reductase complex assembly factor 3 | 1.54233 | - | - |
| Heat shock protein HSP 90-beta | 1.61332 | - | - |
| Protein Fundc2 | 1.65005 | 0.75845 | 0.730198 |
| Bis(5'-nucleosyl)-tetraphosphatase [asymmetrical] | - | 0.765267 | - |
| Protein Mtch2 | - | 1.40781 | - |
| ADP/ATP translocase 1 | - | 1.42962 | - |
| Cytochrome c oxidase subunit 2 | - | 1.44209 | - |
| Nicotinamide nucleotide transhydrogenase | - | 1.37562 | 1.57085 |
| Mitochondrial import inner membrane translocase subunit Tim10 | - | - | 1.32977 |
| Protein Mrpl28 | - | - | 1.33495 |
| Cytochrome c oxidase subunit 7B | - | - | 0.617145 |
| NADH-ubiquinone oxidoreductase chain 5 | - | 1.74921 | - |
| Mitochondrial ribosomal protein S9 | - | - | 1.4717 |
| Annexin | - | - | 0.768474 |
| L-lactate dehydrogenase A chain | - | - | 0.755477 |
| Phytanoyl-CoA dioxygenase, peroxisomal | - | 0.73111 | - |
| Nucleoside diphosphate kinase | - | - | 1.30803 |
| Probable Xaa-Pro aminopeptidase 3 | - | - | 0.601183 |
| Myomesin 2 | - | 1.32486 | - |
| NADH dehydrogenase [ubiquinone] flavoprotein 3 | - | - | 0.755429 |
| NADH dehydrogenase (Ubiquinone) flavoprotein 3-like | - | 1.35897 | - |
| Protein Sco1 | - | - | 1.33068 |
| Acyl carrier protein | - | - | 0.707599 |
| Protein Ppm1k | - | - | 0.730321 |
| BolA-like 1 | - | - | 1.33028 |

**Supplementary Table 2. Mitochondrial proteins annotated by Gene Ontology.**
